# Mechanisms of c-Fos regulation of mTOR signaling via ERα/β in abnormal lipid metabolism of granulosa cells in PCOS

**DOI:** 10.3389/fendo.2025.1587595

**Published:** 2025-08-27

**Authors:** Xinyi Chen, Xiaoxue Shen, Shanyue Guan, Yan Liu, Ning Song, Wenyan Song, Haixia Jin

**Affiliations:** ^1^ Center for Reproductive Medicine, The First Affiliated Hospital of Zhengzhou University, Zhengzhou, China; ^2^ Henan Key Laboratory of Reproduction and Genetics, The First Affiliated Hospital of Zhengzhou University, Zhengzhou, China

**Keywords:** polycystic ovary syndrome, lipid metabolism, c-Fos, estrogen receptor, mTOR, triglycerides

## Abstract

**Objective:**

This study investigates the role of the c-Fos/estrogen receptors (ERs)/mTOR pathway in lipid metabolism in human follicular granulosa cells of individuals with polycystic ovary syndrome (PCOS). Specifically, we aim to determine whether c-Fos targets estrogen receptors (ERα and ERβ) to mediate the mTOR pathway, influencing lipid metabolism, and to identify the key molecular mechanisms involved.

**Methods:**

A PCOS mouse model was established using dehydroepiandrosterone (DHEA), and ovarian tissues were collected from both PCOS and control mice. RT-qPCR and Western blotting were used to measure the expression levels of c-Fos, ERα, ERβ, and mTOR. Follicular fluids were obtained from patients with PCOS and male factor infertility on the day of ovulation. Adenovirus-mediated upregulation of c-Fos was performed in human follicular granulosa cells from male infertility patients, followed by analysis of mRNA and protein levels of c-Fos, ERα, ERβ, and mTOR. Additionally, granulosa cells’ triglyceride (TG) and total cholesterol (TC) levels were assessed. Granulosa cells were cocultured with various concentrations of 17β-estradiol to investigate the effects of estrogen on the pathway.

**Results:**

In ovarian tissues of PCOS mice, mRNA and protein levels of c-Fos were significantly elevated compared to controls, while ERα and ERβ expression was notably reduced. No significant changes were observed in the p-mTOR/mTOR protein ratio. In PCOS patients, c-Fos and p-mTOR/mTOR protein levels were higher than in male factor infertility patients, while ERα levels were lower, with no significant difference in ERβ expression between the two groups. Upregulation of c-Fos in human granulosa cells led to a significant reduction in ERα and ERβ levels, while p-mTOR/mTOR protein levels increased. TG content was elevated in the c-Fos-upregulated group compared to controls, but no significant changes were observed in TC levels. Co-culture of granulosa cells with increasing concentrations of 17β-estradiol resulted in significantly higher ERα and ERβ expression, decreased p-mTOR/mTOR levels, and a reduction in TG content, while TC levels remained unchanged.

**Conclusions:**

These findings suggest that c-Fos may target ERα and ERβ to mediate the mTOR signaling pathway, thereby influencing lipid metabolism in granulosa cells. This novel mechanism provides insights into potential therapeutic strategies for managing PCOS-related metabolic dysfunction.

## 1 Introduction

Polycystic ovary syndrome (PCOS) is a common, lifelong endocrine and metabolic disorder with a highly heterogeneous phenotype. Clinically, PCOS is characterized by ovulatory dysfunction, hyperandrogenism, and polycystic ovaries, often accompanied by insulin resistance and dyslipidemia (1). Obesity has been shown to impair meiotic spindle formation in oocytes and preimplantation embryos, while dyslipidemia significantly affects the onset and progression of PCOS.

Studies indicate that follicular fluid in PCOS patients contains elevated levels of specific fatty acids, including increased total cholesterol (hyper TC), triglycerides (hyper TG), and decreased high-density lipoprotein (low HDL). These lipid abnormalities not only elevate the risk of cardiovascular disease and non-alcoholic fatty liver disease in PCOS patient (2, 3), but they also contribute to ovulatory dysfunction and metabolic disturbances by impacting gonadotropin secretion within the hypothalamic-pituitary-gonadal axis, which may have toxic effects on reproduction. Although assisted reproductive technologies (ART) offer solutions for infertility in PCOS patients, poor oocyte quality remains a significant challenge during *in vitro* fertilization (4).

The formation of follicular fluid, which contributes to the ovarian microenvironment, is influenced by serum components through osmosis and secretion by follicular membranes and granulosa cells. This fluid contains various metabolites, amino acids, hormones, growth factors, and lipids, any of which may affect oocyte developmental capacity when disrupted (5–7). Li et al. found that PCOS patients have abnormal free fatty acids in their follicular fluid, including palmitic acid, arachidonic acid, and stearic acid. The metabolic pathways involved are linked to glycolipid and glycerophospholipid metabolism (8, 9). Additionally, alterations in glycerolipid and glycerophospholipid metabolism in follicular fluid are associated with PCOS (10). In our RNA sequencing analysis of granulosa cells from non-obese PCOS patients and those with tubal factor infertility, differentially expressed genes (DEGs) were primarily related to lipid metabolism, suggesting that abnormal gene expression may impact lipid metabolism in the follicular fluid (11).

Estrogen receptors (ERs), namely ERα and ERβ, expressed on granulosa cells, bind to estradiol (E2) and initiate downstream signaling pathways essential for the normal function of these cells, including the regulation of oocyte maturation (12). These receptors are also closely linked to lipid metabolism (13). ERα has been implicated as a candidate gene for obesity; knockout of ERα in female mice leads to increased fat cell size, number, and elevated cholesterol levels (14). Polymorphisms in ERβ are associated with higher body mass index, elevated serum triglycerides, and increased levels of apolipoprotein B (15). Estrogen receptors ERα and ERβ play distinct but complementary roles in ovarian granulosa cells. ERα is primarily involved in promoting granulosa cell proliferation and modulating the release of pituitary gonadotropins, thereby supporting follicular development and ovulation. Its activity is enhanced by exogenous gonadotropin stimulation, which helps alleviate anovulation. In contrast, ERβ acts as a crucial transcriptional regulator, maintaining granulosa cell differentiation, follicular homeostasis, and ovulatory function. It also participates in steroidogenic gene regulation via phosphorylation-dependent mechanisms and influences ovarian responsiveness to gonadotropins, including modulation of FOXL2 expression (16). Previous studies have shown that expression of ERα and ERβ is significantly downregulated in granulosa cells of PCOS patients (17), yet the relationship between the abnormal expression of these receptors and lipid metabolism in follicular fluid remains unclear.

c-Fos, a key component of the Activator Protein 1 (AP-1) complex, plays a critical role in regulating cell proliferation, differentiation, and apoptosis, and is closely related to reproductive function. ERα can promote the expression of insulin-like growth factor-1, choline acetyltransferase, and collagenase by interacting with AP-1 binding sites (18). Gonadotropins stimulate c-Fos expression in ovarian tissues (19), and c-Fos has been shown to inhibit the transcription of estrogen receptors in breast and cervical cancers (20). Knockdown of c-Fos reduces the expression of all estrogen-regulated genes (21), but the role of c-Fos in regulating ERα and ERβ in granulosa cells remains unexplored.

The mammalian target of rapamycin (mTOR) is a conserved serine/threonine kinase that functions as an anabolic signal activated by various stimuli. mTOR exists in two complexes: mTORC1 and mTORC2 (22). mTORC1, in particular, is involved in lipid and cholesterol metabolism by activating sterol regulatory element-binding protein 1C (SREBP-1C) and peroxisome proliferator-activated receptor α (PPARα), which regulate lipid homeostasis and elevate triglyceride levels. Rapamycin, an mTOR inhibitor, has been shown to prevent or treat anovulatory PCOS by promoting protein synthesis and regulating luteinizing hormone (LH) secretion (23). Furthermore, ERα is associated with mTOR signaling in breast cancer cells, making it a potential therapeutic target (24). However, no studies have examined whether the abnormal expression of ERs in PCOS granulosa cells regulates mTOR and affects lipid metabolism.

PCOS is a multifactorial endocrine-metabolic disorder with a complex pathogenesis that remains incompletely understood. Few studies have focused on the critical role of lipid dysregulation in granulosa cells in the development of PCOS. Therefore, the present study aims to explore whether c-Fos targets ERα/β to regulate lipid metabolism in granulosa cells by mediating the mTOR pathway. Using ovarian tissues from PCOS mice and granulosa cells from PCOS patients, we will investigate the interaction sites and mechanisms between c-Fos, ERα/β, and mTOR, thereby providing a theoretical basis for the early prevention and treatment of PCOS and improving patient outcomes.

## 2 Materials and methods

### 2.1 Human follicular granulosa cells

The study subjects were female patients who underwent *in vitro* fertilization (IVF) treatment at the First Affiliated Hospital of Zhengzhou University between January 2022 and December 2022. Informed consent was obtained from each patient prior to sample collection. Discarded follicular fluid collected during the egg retrieval procedure was used for research purposes on the same day.

#### 2.1.1 Inclusion criteria: PCOS group

Women diagnosed with polycystic ovary syndrome (PCOS) according to the Rotterdam criteria, which require at least two of the following three criteria: 1) Oligo- or anovulation, 2) Clinical and/or biochemical signs of hyperandrogenism, or 3) 1. Polycystic ovaries, with exclusion of other etiologies such as congenital adrenal hyperplasia, androgen-secreting tumors, and Cushing’s syndrome. Control group: Women who meet the following criteria: 1) Infertility due to male factors, 2) Regular menstrual cycle, and 3) Normal ovarian morphology on ultrasound. Human follicular granulosa cells that met the criteria of this group were used for comparison with the PCOS group and subsequent adenovirus overexpression and 17β-estradiol co-culture experiments.

#### 2.1.2 Exclusion criteria

1) Age ≥ 35 years, 2) BMI < 18.5 kg/m² or BMI ≥ 24 kg/m², 3) Dyslipidemia, 4) Other androgen-related hyperplasia and endocrine disorders, 5) Chromosomal abnormalities, and 6) History of endometriosis or ovarian surgery.

Follicular fluid from patients with PCOS treated with the GnRH-ant protocol was used, specifically on days 2 or 3 of the menstrual cycle, when patients were administered recombinant FSH (112.5–300 IU, Puregon, Organon, Netherlands). Adjustments to gonadotropin doses and the addition of FSH and exogenous LH were based on estrogen levels in the follicles. Human chorionic gonadotropin (hCG) (2000 IU, Livzon, China) and recombinant hCG (250 μg, Merck, Italy) were administered when the following criteria were met: one dominant follicle with a diameter ≥ 20 mm, two follicles with a diameter ≥ 18 mm, or three follicles with a diameter ≥ 17 mm. For patients with male-factor infertility, we also use the GnRH antagonist protocol; however, in certain cases, we may adjust to the GnRH agonist long protocol based on the patient’s specific condition. This protocol selection is based on the patient’s ovarian reserve function, previous response to ovarian stimulation, etc., to optimize treatment outcomes and reduce complications.

### 2.2 Experimental animals

Three-week-old female SPF-grade C57BL/6N mice, obtained from Beijing Charles River Experimental Animal Technology Co., Ltd., weighing 8–11 grams, were used in this study.

### 2.3 PCOS mouse model construction

The mice were randomly divided into two groups: a control group and a PCOS group, with 12 mice in each group. After acclimatization to the experimental environment, a PCOS model was induced by subcutaneous injection of dehydroepiandrosterone (DHEA) (Macklin, China) at the nape of the neck. Each mouse in the PCOS group was administered 6 mg of DHEA per 100 grams of body weight daily for 21 consecutive days. The control group received an equal volume of soybean oil daily.

After the treatment period, serum samples were collected by eyeball removal, and serum sex hormones were measured using ELISA kits (Elabscience, China) according to the manufacturer’s instructions. Luteinizing hormone (LH) was measured using a double antibody sandwich method, while testosterone levels were assessed via a competitive binding assay. Anesthesia was induced via intraperitoneal injection at a dose of 40 mg/kg using a 0.7% sodium pentobarbital solution. Following euthanasia by cervical dislocation, ovaries were excised, processed, and embedded in paraffin wax using a tissue embedding machine. Paraffin tissue sections were dewaxed and sealed with a sealing agent.

### 2.4 Isolation and extraction of human follicular granulosa cells

Following the inclusion and exclusion criteria for the study, discarded follicular fluid from patients on the day of egg retrieval was collected into sterile culture bottles and transferred into 50 mL centrifuge tubes. The samples were centrifuged at 4°C, 2000 g for 10 minutes. After centrifugation, 10 mL of sterile PBS buffer was added to the pellet and mixed well. The granulocyte suspension was carefully layered into 10 mL of lymphocyte isolation solution for centrifugation at 4°C, 2500 g for 20 minutes. After centrifugation, the contents were separated into four layers: 1) PBS buffer (top layer), 2) A thin buffy coat lymphocyte layer,3) A cloudy granulosa cell layer, and 4) A bottom erythrocyte layer.

The granulosa cell layer was carefully pipetted into a new centrifuge tube, and a final centrifugation step was performed at 4°C, 1500 g for 10 minutes. After discarding the supernatant, 10 mL of PBS buffer was added, mixed well, and centrifuged again. The resulting pellet contained granulosa cells, which were either used for cell culture or processed for RNA and protein extraction, depending on the requirements of the subsequent experiments.

### 2.5 Adenovirus infection experiments

Granulosa cells were resuspended at a concentration of 1 × 10^6^ cells/mL in complete medium and seeded into 6-well plates, with each well containing cells from the experimental and control groups. Each group was performed in triplicate (three parallel replicates).

In the experimental group, 5 × 10^8^ PFU of c-Fos adenovirus (Genechem, China) was added to each well. In the control group, 5 × 10^8^ PFU of a negative control virus was added to each well. After 8–12 hours of infection, the medium was replaced with fresh complete medium. Fluorescent expression and cell growth were monitored under a microscope at 48 hours post-infection.

After confirming successful infection, total RNA was extracted at 48 hours, and total protein was extracted at 72 hours for further analysis.

### 2.6 17β-estradiol co-culture with human follicular granulosa cells

A 1 mmol/L stock solution of 17β-estradiol (Solarbio, China) was prepared by dissolving 0.01 g of 17β-estradiol in 3.67 mL of dimethyl sulfoxide (DMSO) and stored at -20°C.

After 24 hours of cell attachment, the culture medium was aspirated and replaced with basal medium containing 17β-estradiol at concentrations of 10^-4^ mmol/mL and 10^-5^ mmol/mL. The concentrations of 17β-estradiol (0, 10^-4^ mmol/mL ≈ 10 nm, and 10^-4^ mmol/mL ≈ 100 nm) were selected based on the established *in vitro* literature for granulosa cells, were selected based on the established *in vitro* literature for granulosa cells, providing a physiologically relevant low dose close to follicular fluid levels and a supra-physiological high dose sufficient to elicit robust ER- and mTOR-dependent responses without cytotoxicity, thereby enabling a clear dose–response assessment of E2 on ERα, ERβ, and mTOR expression (25, 26). The control group was treated with an equal volume of DMSO in basal medium. The cells were then incubated for 24 hours. After incubation, the culture medium was discarded, and cells were washed with PBS for subsequent experiments.

### 2.7 RT-qPCR to detect mRNA expression

After RNA extraction, total RNA purity and concentration were assessed using a Nanodrop Ultra-Micro Spectrophotometer (Thermo Fisher, USA) with RNA dissolved in enzyme-free water. The extracted RNA was then used for reverse transcription.

#### 2.7.1 Reverse transcription reaction

The components of the Reverse Transcription Kit (Vazyme R333, China) were thawed on ice and briefly centrifuged to collect them at the bottom of the tube. The following reaction mixture was prepared in a 200 µL enzyme-free centrifuge tube: 4 µl of 5× All-in-one qRT SuperMix, 1 µl of Enzyme Mix, 1 pg to 1 µg of Template RNA, and RNase-free ddH2O to a final volume of 20 µl.

The reaction mixture was thoroughly mixed and incubated in the PCR instrument with the following program: 50°C for 15 minutes, followed by 85°C for 5 seconds.

After the reverse transcription, the cDNA samples were stored temporarily at -80°C.

#### 2.7.2 Quantitative PCR

For qPCR, the components of the Vazyme Q712 Kit (China) were thawed on ice. The following reaction mixture was prepared in a PCR tube: 10 µL of 2× Taq Pro Universal SYBR qPCR Master Mix, 0.4 µL of upstream primer (10 µM), 0.4 µL of downstream primer (10 µM), an appropriate amount of cDNA template, and RNase-free ddH2O to a final volume of 20 µL.

The components were mixed, and the tube was briefly centrifuged to collect the contents at the bottom. The qPCR reaction was run using the following program: 1) Pre-denaturation (1 cycle): 95°C for 30 seconds, 2) Cycling Reaction (40 cycles): 95°C for 10 seconds (denaturation),60°C for 30 seconds (annealing/extension), 3) Melting Curve (1 cycle): 95°C for 15 seconds, 60°C for 60 seconds, 95°C for 15 seconds.

Data collection was performed during the final cycle, and statistical analysis was carried out on the results. Relative mRNA expression was normalized to the housekeeping gene GAPDH, whose stability across treatments was validated by geNorm. The sequences of the PCR primers (provided by Bioengineering, China) are listed in **Table 1**.

**Table 1:** Primer sequences for the gene of interest.

Primer	Species	Direction	Sequence (5’-3’)
GAPDH	Human	Forward primer Reverse primer	TGACCCTCGTTTTGCCATGA GTTGCTGCTGGTTGGAGTTG
c-Fos	Human	Forward primer Reverse primer	CTTCCCAGAAGAGATGTCTGTG TGGGAACAGGAAGTCATCAAAG
ERα	Human	Forward primer Reverse primer	TGTGCCTGGCTAGAGATCCT ATGCGATGAAGTAGAGCCCG
ERβ	Human	Forward primer Reverse primer	CTGTGCCTCTTCTCACAAGGA TGCTCCAAGGGTAGGATGGAC
c-Fos	Mice	Forward primer Reverse primer	TCTCTAGTGCCAACTTTATCCC GAGATAGCTGCTCTACTTTGCC
ERα	Mice	Forward primer Reverse primer	CCCTCCCGCCTTCTACAGGTC ACGGCACAGTAGCGAGTCTCC
ERβ	Mice	Forward primer Reverse primer	TCTCTTCCCAGCAGCAGTCAGTC TCTCCAGCAGCAGGTCGTACAC

### 2.8 Protein extraction and western blot analysis

#### 2.8.1 Protein extraction for concentration determination

Tissues or cells were lysed using an appropriate lysis buffer, and total protein was extracted according to the manufacturer’s protocol of Novigen’s BCA Protein Concentration Assay Kit. The protein concentration was then determined using the BCA method following the kit’s instructions. GAPDH as the internal reference protein, and ensuring that the protein loading on the gel for each sample was 15–20 µg.

#### 2.8.2 Western blot analysis

For Western blotting, the following primary antibodies were used: Anti-c-Fos, Anti-mTOR, Anti-p-mTOR (Abcam, UK), Anti-ERα, Anti-ERβ (Proteintech, China). GAPDH was detected with mouse anti-GAPDH antibody (Cat. No. 60004-1-Ig, Proteintech, China) (RRID: AB_2107436; 1:5,000) as the loading control.

The extracted proteins were separated by SDS-PAGE and transferred onto PVDF membranes. After blocking with 5% non-fat milk in TBST for 1 hour at room temperature, membranes were incubated with the primary antibodies overnight at 4°C. The membranes were then washed with TBST and incubated with appropriate secondary antibodies for 1 hour at room temperature.

Protein bands were visualized using enhanced chemiluminescence (ECL), and the intensity of each band was analyzed using Image J software to quantify the gray values of the proteins of interest.

### 2.9 TG and TC content determination

Triglycerides (TG) and total cholesterol (TC) content were measured according to the instructions for the TG (E-BC-K261-M) and TC (E-BC-K109-M) Colorimetric Test Cassette.

#### 2.9.1 Cell sample preparation

A total of 200 μl of isopropanol was added to the collected cell pellet (2 × 10^6^ cells). The mixture was placed in an ice-water bath, and cells were disrupted using an ultrasonic breaker set at 200W, with 2-second bursts at 3-second intervals for a total of 5 minutes, until the cell pellet was no longer visible.

#### 2.9.2 Centrifugation

The lysate was centrifuged at 10,000 g for 10 minutes at 4°C, and the supernatant was collected and kept on ice for testing.

#### 2.9.3 Mice serum preparation

Mice serum, stored at -80°C, was thawed on ice for direct measurement.

#### 2.9.4 Colorimetric assay

The following procedure was performed for both TG and TC assays: 1) 2.5 μl of double-distilled water, standard solutions, and sample supernatants were added to the designated enzyme-linked immunosorbent assay (ELISA) plate wells. Wells were categorized as blank, standard, or sample wells, and duplicates were prepared for each. 2) Enzyme Working Solution (250 μl) was added to each well to avoid bubble formation. The plate was incubated at 37°C for 10 minutes. 3) After incubation, the optical density (OD) was measured at 510 nm, and the TG and TC concentrations were calculated using the provided standard curve and formula. The catalog numbers and RRIDs for all antibodies and other key reagents are presented in **Table 2**.

**Table 2:** The catalog numbers and RRIDs for antibodies and other relevant reagents.

Designation	Source	Identifiers
Mouse LH ELISA kits	Elabscience, China	E-EL-M3053
QuicKey Pro Mouse T ELISA kits	Elabscience, China	E-OSEL-M0003
Reverse Transcription Kit	Vazyme, China	R333
PCR Kit	Vazyme, China	Q712
Overexpression adenovirus particles	Genechem, China	GV314
17β-estradiol	Solarbio, China	GBW09223
TG	Elabscience, China	E-BC-K261-M
TC	Elabscience, China	E-BC-K109-M
Anti-c-Fos	Abcam, UK	AB_2737414
Anti-mTOR	Abcam, UK	AB_2800465
Anti-p-mTOR	Abcam, UK	AB_2800466
Anti-ERα	Proteintech, China	AB_11042600
Anti-ERβ	Proteintech, China	AB_2102386
Anti-GAPDH	Proteintech, China	AB_2107436
HRP-conjugated Goat Anti-Rabbit IgG(H+L)	Proteintech, China	AB_2722564
HRP-conjugated Goat Anti-Mouse IgG(H+L)	Proteintech, China	AB_2722565

### 2.10 Statistical methods

Data are presented as mean ± standard deviation (SD). All statistical analyses were performed using SPSS version 26.0. The significance of differences between groups was assessed using the independent samples t-test for comparisons between two groups, and one-way analysis of variance (ANOVA) for comparisons among multiple groups. All experiments were performed across 3 to 6 independent biological replicates, with three technical replicates (analytical repeats) per condition in each experiment. The data presented in the figures represent the average values obtained from these independent experiments. In cases where data showed significant outliers attributable to operational error, such values were excluded following verification. Each sample was measured three times per experiment to ensure data reliability. A value of P < 0.05 was considered statistically significant. Asterisks in the figure legends indicate significance: *P < 0.05, **P < 0.01.

## 3 Results

### 3.1 Expression of c-Fos, ERα, ERβ, and mTOR in ovarian tissue of a mouse model of PCOS

To validate the successful establishment of the C57BL/6N mouse PCOS model, we conducted verification from multiple aspects, such as ovarian tissue HE staining, PCOS mouse estrous cycle, weight changes in PCOS mice, and serum LH and T levels in PCOS mice (**Supplementary File S1**). Following successful establishment of the PCOS model in C57BL/6N mice, pregnant mare serum gonadotropin (PMSG) was administered intraperitoneally to both the PCOS and control groups. After 48 hours, human chorionic gonadotropin (hCG) was injected intraperitoneally, and cumulus-oocyte complexes were collected. Granulosa cells were then carefully isolated from the follicles under a stereomicroscope, allowing for cell-type-specific analyses.

#### 3.1.1 Serum LH and testosterone levels

**Figure 1:**
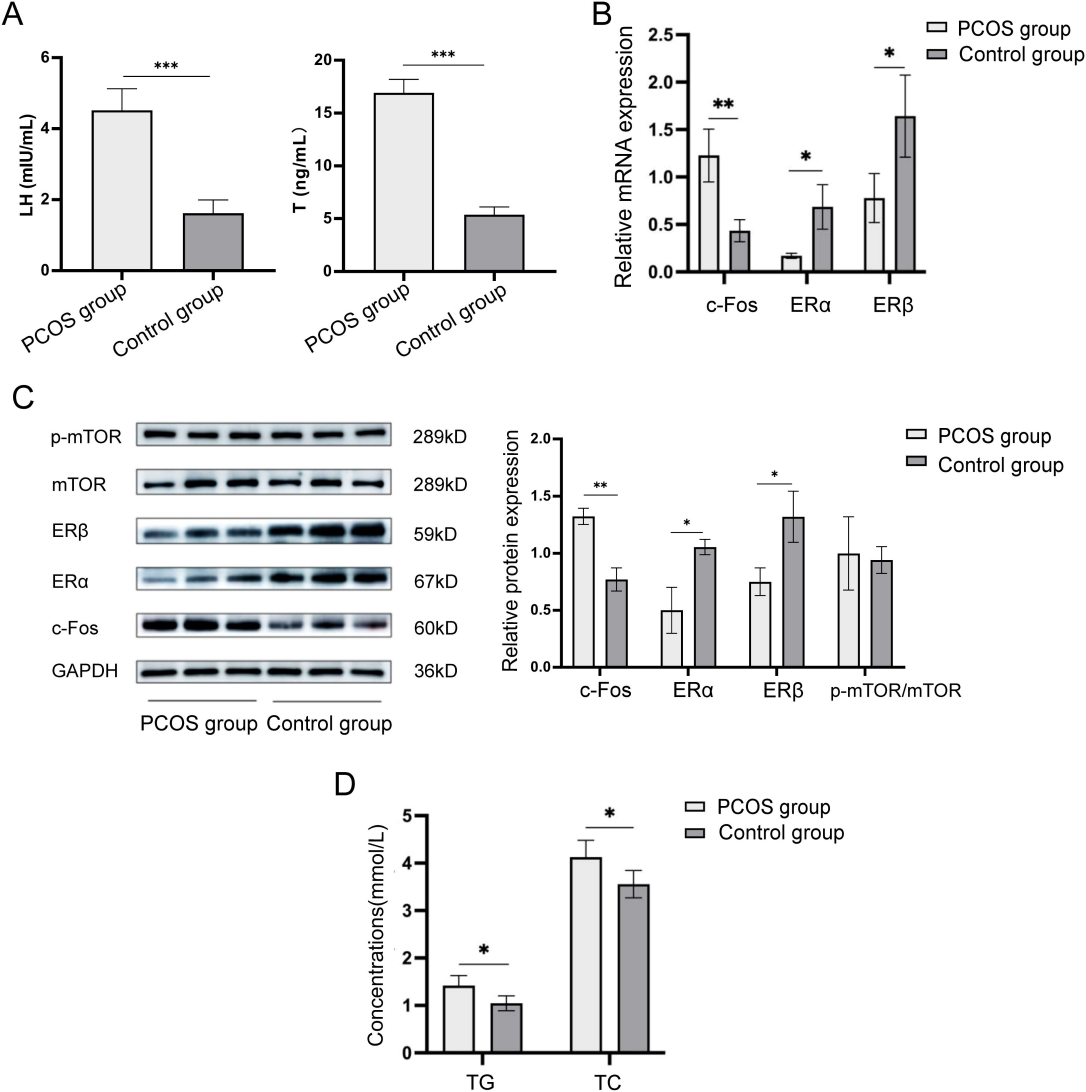
Expression of c-Fos, ERα, ERβ, and mTOR in ovarian tissue of mice models in the PCOS group and control group. **(A)** Comparison of serum LH and T levels in mice in the PCOS and control groups. **(B)** Relative mRNA expression levels of c-Fos, ERα, and ERβ in ovarian tissues of mice in the PCOS group and control group. **(C)** Relative protein expression levels of c-Fos, ERα, ERβ, and p-mTOR/mTOR in ovarian tissues of mice in the PCOS group and control group. **(D)** Comparison of serum TG and TC levels in ovarian tissues of mice in the PCOS group and control group. *P<0.05; **P<0.01, ***P<0.001.

The serum levels of luteinizing hormone (LH) and testosterone (T) were measured using ELISA kits. As shown in **Figure 1A**, the results indicated significant differences between the PCOS and control groups. Specifically, the serum LH level in the PCOS group was significantly higher than in the control group (4.52 ± 0.61 mIU/mL vs. 1.62 ± 0.37 mIU/mL, p < 0.001), and similarly, the serum T level in the PCOS group was also significantly elevated (16.92 ± 1.26 ng/mL vs. 5.37 ± 0.74 ng/mL, p < 0.001). Both differences were statistically significant.

#### 3.1.2 mRNA expression of c-Fos, ERα, and ERβ in ovarian tissue

The mRNA expression levels of c-Fos, ERα, and ERβ in ovarian tissues from the PCOS and control mice were measured using RT-qPCR. The results showed that the mRNA expression of c-Fos in the PCOS group was significantly higher than in the control group (1.228 ± 0.278 vs. 0.434 ± 0.117, p = 0.004). In contrast, the mRNA expression of ERα was significantly lower in the PCOS group compared to the control group (0.170 ± 0.027 vs. 0.686 ± 0.234, p = 0.03), and similarly, ERβ expression was also significantly reduced (0.778 ± 0.258 vs. 1.642 ± 0.433, p = 0.02) (**Figure 1B**).

#### 3.1.3 Protein expression of c-Fos, ERα, ERβ, and mTOR

Western blot analysis was performed to evaluate the protein expression levels of c-Fos, ERα, ERβ, and mTOR in ovarian tissues. As shown in **Figure 1C**, the protein expression of c-Fos in the PCOS group was significantly higher than in the control group (1.323 ± 0.070 vs. 0.770 ± 0.101, p = 0.002). The protein levels of ERα (0.500 ± 0.205 vs. 1.053 ± 0.067, p = 0.011) and ERβ (0.750 ± 0.123 vs. 1.320 ± 0.219, p = 0.017) were significantly lower in the PCOS group compared to the control group. However, there was no significant difference in the protein expression of p-mTOR/mTOR between the two groups (1.000 ± 0.322 vs. 0.940 ± 0.115, p = 0.776).

#### 3.1.4 Serum TG and TC levels

The serum levels of triglycerides (TG) and total cholesterol (TC) were assessed using colorimetric kits. The results showed that the serum TG content was significantly higher in the PCOS group compared to the control group (1.42 ± 0.21 mmol/L vs. 1.05 ± 0.16 mmol/L, p = 0.035). Similarly, the serum TC content was also significantly elevated in the PCOS group (4.13 ± 0.36 mmol/L vs. 3.56 ± 0.29 mmol/L, p = 0.046) (**Figure 1D**).

### 3.2 Expression of c-Fos, ERα, ERβ, and mTOR in granulosa cells of PCOS patients

This study collected 30 cases each of follicular fluid granulosa cells from patients with PCOS (PCOS group) and patients with male factor infertility (control group). Blood samples were collected on days 2–4 of the menstrual cycle. The comparison of baseline data between the two groups is shown in **Table 3**. There were no significant differences between the two groups in terms of age, BMI, and baseline follicle-stimulating hormone (FSH) levels. The baseline LH levels in the PCOS group were significantly higher than those in the control group (12.18 ± 6.03 mIU/ml vs. 5.55 ± 2.22 mIU/ml, P = 0.001), and the baseline T levels in the PCOS group were significantly higher than those in the control group (1.06 ± 0.85 ng/ml vs. 0.35 ± 0.20 ng/ml, P = 0.009).

**Table 3:** Comparison of baseline data between the PCOS group and the control group patients.

Variables	PCOS group(n=30)	Control group(n=30)	P
Age (y)	26.64 ± 3.56	28.69 ± 3.37	0.161
BMI (kg/m^2^)	22.06 ± 1.36	20.78 ± 1.83	0.069
Basal LH (mIU/ml)	12.18 ± 6.03	5.55 ± 2.22	0.001**
Basal FSH (mIU/ml)	5.53 ± 1.29	6.19 ± 1.59	0.282
Basal T (ng/ml)	1.06 ± 0.85	0.35 ± 0.20	0.009**

**P<0.01.

**Figure 2:**
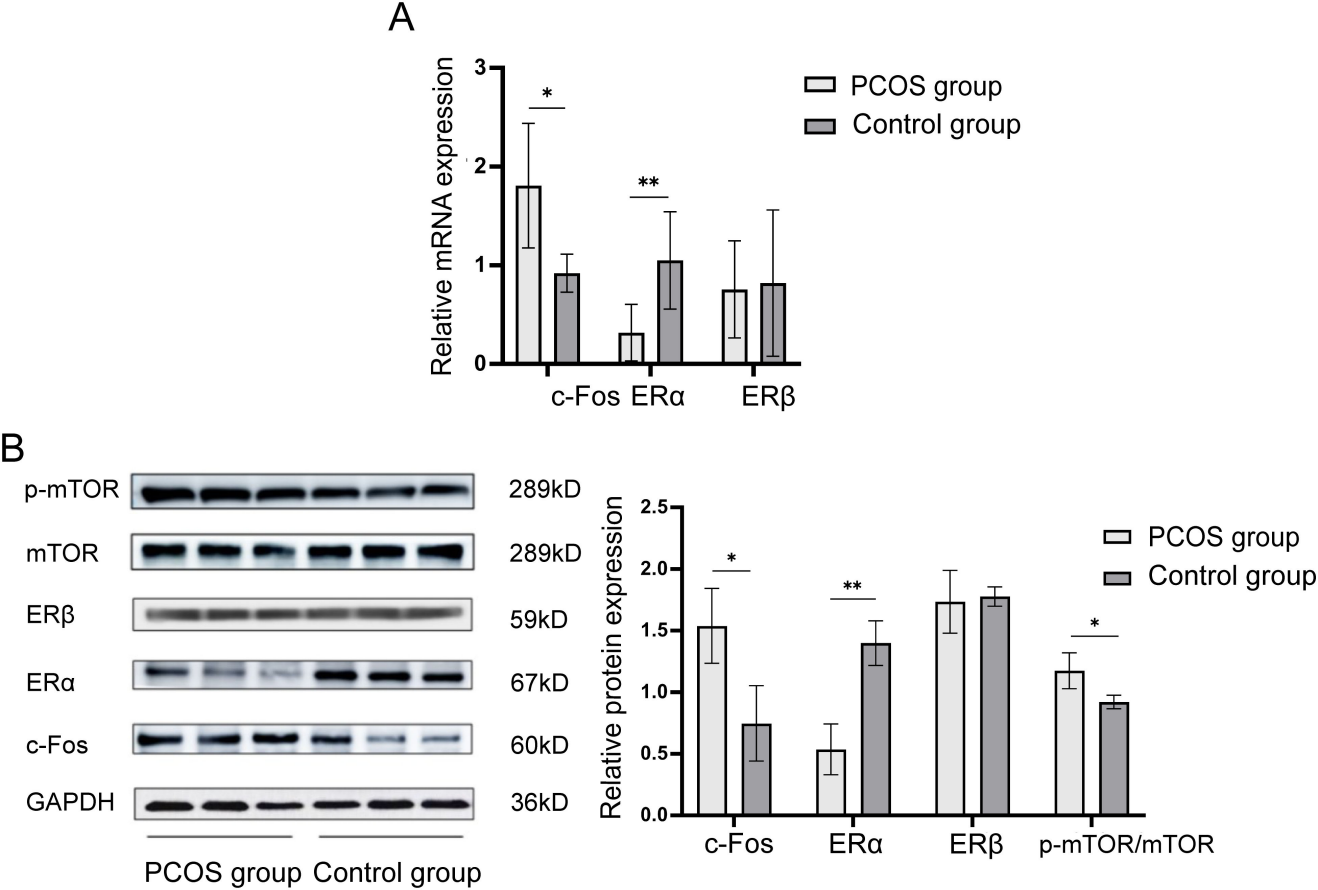
Expression levels of c-Fos, ERα, ERβ, and mTOR in granulosa cells of PCOS patients and control group. **(A)** Relative mRNA expression levels of c-Fos, ERα, and ERβ in granulosa cells of the PCOS group and the control group. **(B)** Relative protein expression levels of c-Fos, ERα, ERβ, and mTOR in granulosa cells of PCOS group and control group. *P<0.05; **P<0.01.

The mRNA expression levels of c-Fos, ERα, and ERβ in granulosa cells from the follicular fluid of both PCOS and control groups were measured. As shown in **Figure 2A**, the relative mRNA expression of c-Fos in the PCOS group was significantly higher than in the control group (1.898 ± 0.660 vs. 0.993 ± 0.247, P = 0.012). In contrast, the relative mRNA expression of ERα was significantly lower in the PCOS group (0.209 ± 0.119 vs. 1.019 ± 0.449, P = 0.004), while no significant difference in the mRNA expression of ERβ was observed between the two groups (0.839 ± 0.501 vs. 0.741 ± 0.691, P = 0.798).

Western blot analysis was performed to assess the protein expression levels of c-Fos, ERα, ERβ, and mTOR in granulosa cells. The results revealed that c-Fos protein expression was significantly higher in the PCOS group compared to controls (1.540 ± 0.304 vs. 0.743 ± 0.309, P = 0.033), while ERα expression was significantly lower in the PCOS group (0.533 ± 0.206 vs. 1.400 ± 0.185, P = 0.006). No significant difference was found for ERβ (1.733 ± 0.257 vs. 1.780 ± 0.079, P = 0.779). Additionally, the ratio of p-mTOR/mTOR expression was higher in the PCOS group than in the control group (1.173 ± 0.145 vs. 0.920 ± 0.052, P = 0.046). These findings suggest that c-Fos and p-mTOR/mTOR are elevated in granulosa cells of PCOS patients, while ERα expression is significantly reduced (**Figure 2B**).

### 3.3 Overexpression of c-Fos in human follicular granulosa cells using adenovirus

Our results indicate that the expression of c-Fos is lower in granulosa cells of patients with male factor infertility alone compared to those in PCOS patients. To investigate the effects of c-Fos overexpression, adenovirus was used to upregulate c-Fos in granulosa cells from patients with male factor infertility. The expression of the target gene and changes in lipid metabolism in human follicular granulosa cells were subsequently observed.

**Figure 3:**
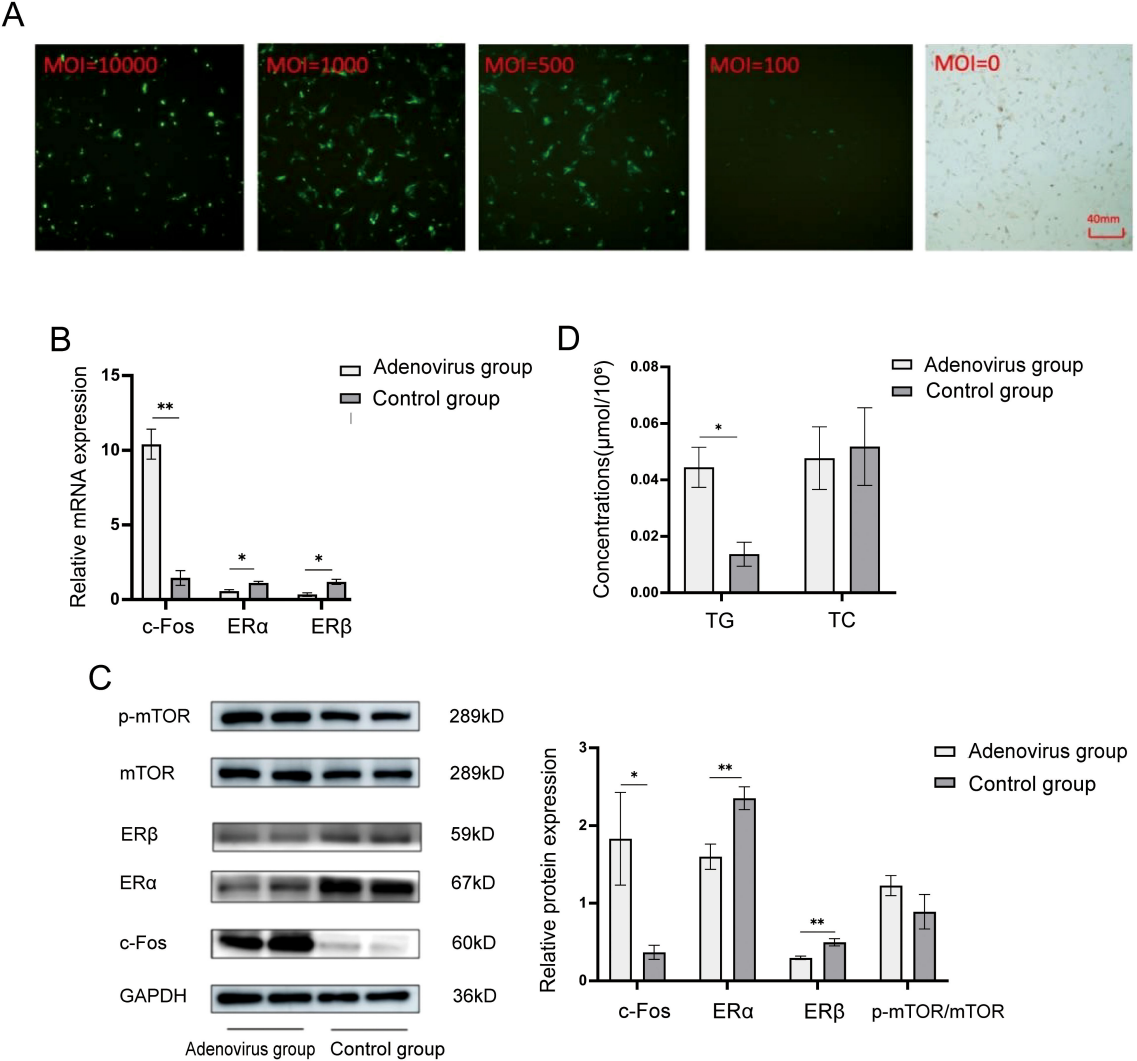
ERα, ERβ, and mTOR were expressed in adenovirus-infected human follicular granulosa cells with a value of MOI=500 following c-Fos overexpression. **(A)** Fluorescence of adenovirus-infected human follicular granulosa cells under different MOI conditions under fluorescence microscopy (40x microscope). **(B)** Relative mRNA expression of intracellular c-Fos, ERα, and ERβ after infection. **(C)** Relative protein expression levels of intracellular c-Fos, ERα, ERβ, and mTOR after infection. **(D)** A colorimetric assay detected TG and TC content in cells overexpressing c-Fos. *P<0.05; **P<0.01.

Preliminary experiments were performed to determine the optimal viral titer for human granulosa cells. A range of MOIs (100–10,000) was tested, and both transduction efficiency and cell morphology were continuously monitored under a fluorescence microscope. As shown in **Figure 3A**, efficiency increased with rising MOI; however, overt cell death appeared at MOI 10,000, whereas the increment in efficiency at MOI 1,000 was only marginal compared with MOI 500. Consistent with the principle of employing the lowest effective viral dose to minimize cellular damage, MOI 500—yielding ~80% transduction with intact cell integrity—was selected for all subsequent infections.

The infection efficiency of c-Fos overexpression using MOI = 500 was evaluated by RT-qPCR. As shown in **Figure 3B**, the relative mRNA expression of c-Fos in the overexpression group was significantly higher than in the control group (10.407 ± 1.007 vs. 1.454 ± 0.494, P = 0.004). Conversely, the mRNA levels of ERα (0.570 ± 0.102 vs. 1.116 ± 0.115, P = 0.04) and ERβ (0.338 ± 0.122 vs. 1.196 ± 0.174, P = 0.02) were significantly decreased in the overexpression group.

Western blot analysis was performed to assess the protein expression levels of c-Fos, ERα, ERβ, and mTOR following adenovirus-mediated overexpression of c-Fos in human follicular granulosa cells. As shown in **Figure 3C**, the relative protein expression of c-Fos was significantly increased in the overexpression group (1.830 ± 0.597 vs. 0.367 ± 0.091, P = 0.014). The protein levels of ERα (1.600 ± 0.163 vs. 2.352 ± 0.147, P = 0.004) and ERβ (0.296 ± 0.022 vs. 0.496 ± 0.047, P = 0.003) were significantly reduced. Although the protein expression of p-mTOR/mTOR was upregulated in the overexpression group (1.227 ± 0.129 vs. 0.890 ± 0.222), the difference was not statistically significant (P = 0.085). These results suggest that upregulation of c-Fos inhibits the expression of ERα and ERβ, and leads to a trend of increased p-mTOR/mTOR expression, though the change was not significant.

To examine the impact of c-Fos overexpression on lipid metabolism, the intracellular content of TG and TC was measured using commercial kits. As shown in **Figure 3D**, the intracellular TG content was significantly increased after c-Fos overexpression in human follicular granulosa cells (0.0444 ± 0.0071 μmol/10^6^ vs. 0.0137 ± 0.0042 μmol/10^6^, P < 0.001). However, no significant change in TC content was observed (0.0477 ± 0.0111 μmol/10^6^ vs. 0.0518 ± 0.0137 μmol/10^6^, P = 0.466). These results indicate that c-Fos upregulation increases the intracellular TG content in human follicular granulosa cells but does not affect TC content.

### 3.4 Expression levels of ERα, ERβ, and mTOR in human follicular granulosa cells co-cultured with 17β-estradiol

**Figure 4:**
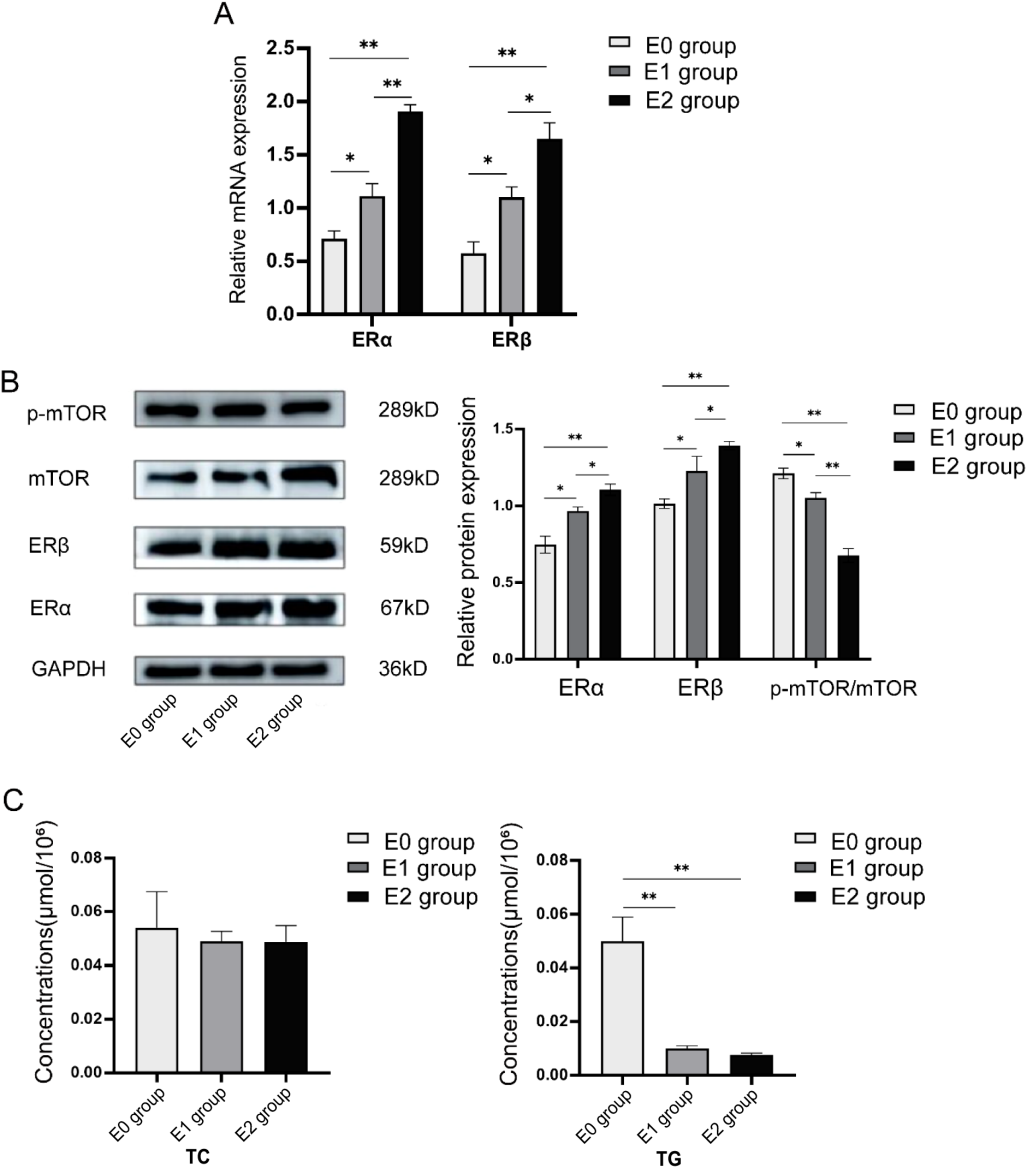
Expression levels of c-Fos, ERα, ERβ, and mTOR in granulosa cells of PCOS patients after 24h of co-culture with human follicular granulosa cells using different concentrations of 17β-estradiol [E0 group (no estradiol), E1 group (10^-5^ mmol/mL), and E2 group (10^-4^ mmol/mL)]. **(A)** After 24 hours of co-culture, RT-qPCR was used to detect the relative mRNA expression of ERα and ERβ in cells after infection. **(B)** After 24 hours of co-incubation, Western blot was used to detect the protein expression levels of ERα, ERβ, and mTOR in cells after infection. **(C)** After 24 hours of co-incubation, a Colorimetric assay was used to detect the content of TG and TC in human follicular granulosa cells in each group. *P<0.05; **P<0.01.

Different concentrations of 17β-estradiol were added to the culture medium of human follicular granulosa cells, and the expression levels of ERα, ERβ, and mTOR were measured after 24 hours of co-culture. The groups included the E0 group (no estradiol), E1 group (10^-5^ mmol/mL), and E2 group (10^-4^ mmol/mL). As shown in **Figure 4A**, the relative mRNA expression of ERα and ERβ increased in a dose-dependent manner with the concentration of 17β-estradiol after 24 hours of incubation. Statistically significant differences were observed in the relative mRNA expression levels of ERα between the groups: E0 vs. E1: 0.713 ± 0.073 vs. 1.113 ± 0.115, P = 0.01; E0 vs. E2: 0.713 ± 0.073 vs. 1.910 ± 0.061, P = 0.004; and E1 vs. E2: 1.113 ± 0.115 vs. 1.901±0.061, P = 0.006. Similarly, significant differences were observed in the relative mRNA expression levels of ERβ: E0 vs. E1: 0.576 ± 0.106 vs. 1.103 ± 0.096, P = 0.02; E0 vs. E2: 0.576 ± 0.106 vs. 1.650 ± 0.151, P = 0.003; and E1 vs. E2: 1.103 ± 0.096 vs. 1.650 ± 0.151, P = 0.01.

Western blot analysis was used to determine the protein expression levels of ERα, ERβ, and mTOR in the three groups. As shown in **Figure 4B**, the protein expression of ERα significantly increased with higher 17β-estradiol concentrations, with statistically significant differences between groups: E0 vs. E1: 0.747 ± 0.055 vs. 0.967 ± 0.025, P = 0.01; E0 vs. E2: 0.747 ± 0.055 vs. 1.103 ± 0.038, P = 0.001; and E1 vs. E2: 0.967 ± 0.025 vs. 1.103 ± 0.038, P = 0.03. Similarly, the expression of ERβ increased with 17β-estradiol treatment: E0 vs. E1: 1.013 ± 0.032 vs. 1.227 ± 0.095, P = 0.04; E0 vs. E2: 1.013 ± 0.032 vs. 1.393 ± 0.025, P = 0.003; and E1 vs. E2: 1.227 ± 0.095 vs. 1.393 ± 0.025, P = 0.014. In contrast, the protein expression of p-mTOR/mTOR decreased with increasing estradiol concentration: E0 vs. E1: 1.210 ± 0.036 vs. 1.050 ± 0.036, P = 0.02; E0 vs. E2: 1.210 ± 0.036 vs. 0.677 ± 0.045, P = 0.004; and E1 vs. E2: 1.050 ± 0.036 vs. 0.677 ± 0.045, P = 0.006. These results suggest that stimulation with estradiol led to upregulation of ERα and ERβ, while downregulating p-mTOR/mTOR protein expression.

Next, changes in intracellular TG and TC contents were examined following stimulation with 17β-estradiol. As shown in **Figure 4C**, intracellular TG content decreased significantly in both the E1 and E2 groups compared to the E0 group: E1 vs. E0: 0.0100 ± 0.0010 μmol/10^6^ vs. 0.0500 ± 0.0089 μmol/10^6^, P < 0.001; E2 vs. E0: 0.0077 ± 0.0006 μmol/10^6^ vs. 0.0500 ± 0.0089 μmol/10^6^, P < 0.001. There was no significant difference in TG content between the E1 and E2 groups (0.0100 ± 0.0010 μmol/ 10^6^ vs. 0.0077 ± 0.0006 μmol/10^6^, P = 0.601). However, no significant change in TC content was observed among the three groups (E0 vs. E1: 0.0540 ± 0.0135 μmol/10^6^ vs. 0.0490 ± 0.0036 μmol/10^6^, P = 0.512; E0 vs. E2: 0.0540 ± 0.0135 μmol/10^6^ vs. 0.0487 ± 0.0061 μmol/10^6^, P = 0.485; E1 vs. E2: 0.0490 ± 0.0036 μmol/10^6^ vs. 0.0487 ± 0.0061 μmol/10^6^, P = 0.964).

In conclusion, the results suggest that the upregulation of ERα and ERβ expression in human follicular granulosa cells, induced by 17β-estradiol, leads to a significant reduction in intracellular TG content, with no observed effect on TC content.

## 4 Discussion

This study explored the molecular mechanisms through which c-Fos regulates the ERα/β pathway in granulosa cells of polycystic ovary syndrome (PCOS) with abnormal lipid metabolism. In the DHEA-induced ovarian tissues of PCOS mice, we observed elevated expression of c-Fos, accompanied by a marked reduction in ERα and ERβ expression. Notably, the expression of c-Fos was significantly higher in the PCOS group compared to the control, while ERα levels were significantly lower.

Further investigations into the effect of c-Fos on lipid metabolism in human follicular granulosa cells revealed that overexpression of c-Fos led to significant downregulation of ERα and ERβ, with a concomitant increase in the expression of p-mTOR/mTOR. Additionally, intracellular TG levels were significantly elevated in c-Fos overexpressing cells compared to the negative control group. These findings suggest that c-Fos may disrupt normal lipid metabolism in granulosa cells, potentially through alterations in ERα/β signaling and mTOR pathways.

Moreover, stimulation of human granulosa cells with 17β-estradiol, a known activator of ERα/β, resulted in increased expression of both ERα and ERβ, along with a reduction in p-mTOR/mTOR expression. Correspondingly, intracellular TG levels decreased, suggesting that ERα/β activation might counteract the lipid dysregulation induced by c-Fos overexpression. These results imply that ERα/β signaling plays a critical role in mediating the effect of c-Fos on lipid metabolism in granulosa cells of PCOS patients.

In summary, our study suggests that c-Fos may influence lipid metabolism in PCOS granulosa cells by modulating the ERα/β pathway and mTOR signaling. This highlights a potential mechanism by which c-Fos contributes to the metabolic abnormalities observed in PCOS, and underscores the importance of ERα/β signaling in regulating lipid homeostasis in ovarian cells.

The c-Fos gene, when activated in the nucleus, synthesizes the Fos protein, which forms a transcriptionally active heterodimer with the Jun protein. This dimer regulates various intracellular activities. In this study, we observed that the mRNA and protein expression levels of c-Fos were significantly higher in both the ovarian tissues of PCOS mice and the granulosa cells of PCOS patients, compared to the control group. Previous studies have reported that immunohistochemistry of human follicular granulosa cells reveals prominent staining in the granulosa cell layer, while the follicular membrane cell layer shows no such staining. Inhibition of c-Fos expression has been associated with increased levels of 17α-hydroxylase and enhanced androgen synthesis (27, 28), suggesting that c-Fos may play a critical role in the hyperandrogenism characteristic of PCOS. Moreover, sequencing of cumulus granulosa cells from PCOS patients by Hao et al. revealed abnormal expression of c-Fos (29), which aligns with our findings and further supports the involvement of c-Fos in the pathogenesis of PCOS.

Additionally, we overexpressed c-Fos in human follicular granulosa cells and observed a significant elevation in intracellular TG content. This suggests that c-Fos may be involved in lipid metabolism within granulosa cells. It has been established that c-Fos regulates phospholipid synthesis and lipid metabolism in various tissues. In liver tissue, overexpression of c-Fos activates the peroxisome proliferator-activated receptor γ (PPARγ) signaling pathway, leading to increased lipid accumulation (30). Similarly, in the central nervous system, c-Fos promotes lipid synthesis in neuronal cells and aids in neuronal differentiation (31). Furthermore, studies have shown that oxidized LDL can enhance the transcriptional activity of c-Fos, increasing lipid uptake, stimulating foam cell proliferation, and accelerating atherosclerosis development (32). These findings indicate that c-Fos may play an essential role in lipid accumulation, potentially contributing to the metabolic abnormalities observed in PCOS.

In this study, we found that the expression of ERα and ERβ in ovarian tissue from PCOS mice was significantly reduced. In PCOS patients, ERα expression in granulosa cells was significantly reduced, while ERβ expression remained unchanged. However, in another study, it was shown that in patients undergoing IVF treatment, ERα and ERβ expression in granulosa cells from PCOS patients were significantly downregulated compared to patients with male-factor infertility alone (33). The intrinsic complex regulatory mechanisms, optimizing experimental designs, measuring hormone levels in samples, and analyzing their effects on ERα and ERβ mRNA expression, as well as investigating potential signaling pathways influencing ERα and ERβ mRNA expression, may help elucidate these mechanisms.

The E2-ERs signaling pathway plays a crucial role in maintaining the normal function of granulosa cells and regulating oocyte maturation (12). Studies in adult mice have shown that ERα knockout leads to infertility, with multiple hemorrhagic or cystic follicles in the ovaries and an increase in steroid synthesis capacity. In contrast, adult mice with ERβ knockout display reduced fertility, a sustained decrease in growing follicles, and a diminished corpus luteum in the ovaries. These findings suggest that the development of PCOS may be linked to the reduced expression of ERα and ERβ in granulosa cells. Reports indicate that overexpression of c-Fos in breast cancer cell lines can inhibit the transcription of ERα and ERβ and effectively suppress the activation of genes containing estrogen response elements. This antagonistic effect may be mediated by protein-protein interactions (34). In our study, we observed that the expression of both ERα and ERβ decreased following the overexpression of c-Fos in human follicular granulosa cells, indicating that c-Fos may inhibit ERα/β transcription in these cells. However, the specific antagonistic sites of c-Fos on ERα/β require further investigation.

Furthermore, stimulation of human follicular granulosa cells with 17β-estradiol resulted in increased expression of ERα and ERβ, accompanied by a decrease in intracellular TG content. This finding provides preliminary evidence that upregulation of ERα and ERβ in human granulosa cells can regulate TG production. Estrogen regulates cellular metabolic homeostasis and lipid metabolism through its action on ERα and ERβ. For instance, in mice with ERα knockout, an increase in body fat and adipocyte size has been reported (35). In adipocytes, upregulation of ERα is associated with reduced intracellular TG accumulation and decreased expression of lipoprotein lipase (36). Additionally, treatment with ERβ agonists in obese mice, induced by high-fat diet and oophorectomy, led to weight loss, reduced serum TC, leptin, glucose levels, and decreased fat accumulation (37).

In addition, we observed that the expression levels of p-mTOR/mTOR were elevated in follicular granulosa cells of PCOS patients. The mTOR signaling pathway plays a crucial role in various aspects of female reproduction, including follicular development, oocyte meiosis, ovarian aging, endometrial changes, and embryonic development (38). Aberrant activation of the mTOR pathway has been shown to be a key factor in the initiation and progression of PCOS (39–41). Studies have demonstrated that excessive insulin can induce granulosa cell apoptosis and activate the AKT/mTOR pathway (42), suggesting a close relationship between the mTOR pathway and insulin resistance in PCOS. Moreover, the mTOR pathway may contribute to the imbalance between cell proliferation and apoptosis in granulosa cells of PCOS patients (43). Previous studies have shown that regulating the mTOR signaling pathway can influence Bcl-2/Bax-induced apoptosis (44).

In this study, we found that the expression of p-mTOR/m-TOR decreased significantly after stimulation of ERα and ERβ expression in human follicular granulosa cells, suggesting a potential regulatory interaction between the ERα/β and mTOR pathways. In ERα-positive breast cancer, inhibition of the ERα-mTOR pathway is an effective therapeutic strategy (24, 45). Upregulation of ERα in PCOS mice inhibits the PI3K/AKT/mTOR pathway, downregulates apoptotic proteins, and improves granulosa cell apoptosis (46). Similarly, our findings showed that after the expression of ERα and ERβ increased in human follicular granulosa cells, the expression of p-mTOR/mTOR decreased, and intracellular TG content decreased. These results suggest that upregulation of ERα and ERβ in granulosa cells may reduce TG synthesis, potentially through inhibition of the mTOR pathway. This study speculates that ERα/β may indirectly influence mTOR activity in human ovarian granulosa cells by regulating upstream signaling molecules of the mTOR pathway, such as the PI3K/AKT pathway. This regulatory mechanism may be closely related to follicular development and ovulation processes, providing new insights into the molecular mechanisms underlying follicular development. It also explains part of the cause of lipid metabolism disorders in PCOS granulosa cells.

In addition, we observed that c-Fos overexpression down-regulated ERα and ERβ and slightly elevated the p-mTOR/mTOR ratio, yet these alterations did not achieve statistical significance. Limited biological replicates, intrinsic heterogeneity among granulosa cells and follicular stages, and cell-cycle-dependent fluctuations in pathway activity render these findings preliminary; they therefore do not support definitive conclusions and warrant further mechanistic investigation.

The mTOR pathway is a major regulator of cellular metabolism, driving anabolic processes such as protein, lipid, cholesterol, and nucleotide synthesis (47, 48). Studies have demonstrated that mTORC1, a complex of mTOR, regulates fatty acid synthesis by controlling the nuclear translocation of SREBP1, a key transcription factor. Upon entering the nucleus, SREBP1 activates the transcription of genes like FASN and ACC, which are essential for palmitic acid synthesis (49, 50). mTORC1 also regulates adipogenesis by modulating FASN and ACC expression (51). Additionally, mTORC2, another complex of mTOR, plays a role in adipogenesis, regulating the expression of carbohydrate response element-binding proteins in adipose tissue. In the liver, mTORC2 regulates SREBP1, and in Rictor-knockout mice, reduced levels of SREBP1 result in lower expression of FASN and ACC, leading to reduced TG accumulation.

In our study, the expression levels of p-mTOR/mTOR were higher in the follicular granulosa cells of PCOS patients compared to those of patients with male factor infertility. This increase in p-mTOR/mTOR expression in granulosa cells correlated with elevated intracellular TG content, suggesting that the abnormal mTOR expression could disrupt intracellular TG metabolism, potentially impacting oocyte quality in PCOS patients. TG (triglycerides) is an aliphatic molecule composed of glycerol and three long-chain fatty acid molecules. It is a major component of animal fats, low-density lipoprotein (LDL), and very low-density lipoprotein (VLDL). Serving as both an energy source and a transport carrier for fatty acids, TG plays a critical role in lipid metabolism (52).

Imbalance in TG metabolism can significantly affect oocyte quality. TG is catabolized by lipases in granulosa cells, producing free fatty acids, which are then metabolized in the mitochondria via β-oxidation to generate ATP. Inhibition of β-oxidation impairs oocyte maturation and embryonic development (53). Studies have shown that the release of fatty acids increases when intracellular TG breakdown is enhanced, helping regulate adipogenesis and lipolysis, maintain lipid homeostasis, and limit reactive oxygen species (ROS) production, ultimately improving oocyte quality (54). On the other hand, overactivation of mTORC1 induces the expression of adipogenic and lipoprotein genes, such as apolipoprotein B, leading to elevated TG levels (55). mTORC1 also inhibits the hydrolysis of TG into free fatty acids by triglyceride lipase and suppresses fatty acid oxidation by controlling the entry of free fatty acids into mitochondria for β-oxidation (56). Consequently, in PCOS granulosa cells, the imbalance in TG metabolism may affect oocyte quality through the mTOR pathway, although the precise molecular mechanisms require further investigation.

High expression of c-Fos, low expression of ER, and elevated p-mTOR levels may serve as combined biomarkers for metabolic subtypes of PCOS. At the therapeutic level, targeting inhibition of c-Fos or mTOR, or selective activation of ER, may represent new strategies for improving ovulatory function and associated metabolic disorders in PCOS. Future studies could further investigate the expression patterns of c-Fos in different PCOS subtypes and their relationship with metabolic syndrome components, including insulin resistance and inflammatory responses. Additionally, developing specific c-Fos inhibitors or ER agonists and validating their efficacy in animal models and clinical samples could provide new theoretical foundations and intervention targets for the precision treatment of PCOS.

Furthermore, to extend our current observations, future studies will employ siRNA-mediated c-Fos knockdown in cultured primary human granulosa cells and systematically evaluate the resulting changes in ERα/β expression, PI3K/AKT/mTOR signaling activity, and key lipid-metabolism indices such as triglyceride and cholesterol levels. By examining whether c-Fos suppression can rescue the aberrant lipid profile characteristic of PCOS models, we aim to elucidate the molecular mechanisms through which c-Fos governs granulosa cell dysfunction and to provide a theoretical basis for novel therapeutic strategies targeting lipid dysregulation in reproductive endocrinology.

In conclusion, the ERα/β and mTOR pathways are critical regulators of granulosa cell function, yet their roles in granulosa cell lipid metabolism remain understudied. In this research, we found that in granulosa cells from PCOS patients, ERα expression was down-regulated, while p-mTOR/mTOR expression was up-regulated. Additionally, c-Fos, a potential upstream regulator, was found to be up-regulated in these cells. Upon adenoviral overexpression of c-Fos in human follicular granulosa cells, we observed a significant reduction in ERα and ERβ expression, accompanied by an increase in p-mTOR/mTOR expression and elevated intracellular TG levels. Conversely, stimulation of ERα and ERβ expression led to a reduction in p-mTOR/mTOR expression and a decrease in TG content. These findings suggest that c-Fos may target ERα and ERβ in granulosa cells of PCOS patients, modulating the mTOR pathway and contributing to lipid metabolic dysfunction in these cells.

## 5 Conclusion

Our research found that the significantly elevated c-Fos expression in the ovarian tissue of PCOS mice, by ERα and ERβ expression was markedly downregulated. Additionally, serum triglyceride (TG) and total cholesterol (TC) levels were significantly higher than those in the control group. Further analysis of clinical samples revealed that, compared to patients with male-factor infertility, PCOS patients exhibited elevated c-Fos and p-mTOR/mTOR expression in granulosa cells, while ERα expression was downregulated, and ERβ expression showed no significant changes. Functional experimental results showed that upregulating c-Fos in human granulosa cells significantly reduced ERα and ERβ expression, activated the mTOR signaling pathway, and promoted TG production, while having no significant effect on TC. This study suggests that c-Fos influences lipid metabolism by regulating the ERα/β-mTOR pathway.

These findings provide new insights and approaches for the prevention and treatment of PCOS. Future studies may further explore the expression patterns of c-Fos in different subtypes of PCOS and its association with metabolic syndrome components such as insulin resistance and inflammatory responses, and develop specific c-Fos inhibitors or ERα agonists to provide new theoretical basis and intervention targets for the precision treatment of PCOS.

## Data Availability

The data analyzed in this study is subject to the following licenses/restrictions: The data underlying this article will be shared on reasonable request to the corresponding author. Requests to access these datasets should be directed to Haixia Jin, Email: jinhx@zzu.edu.cn.
