# Supplementary material for: Mechanisms of c-Fos regulation of mTOR signaling via ERα/β in abnormal lipid metabolism of granulosa cells in PCOS
Source: Front Endocrinol (Lausanne). 2025 Aug 27;16:1587595. doi: 10.3389/fendo.2025.1587595 (PMC12421171; doi:10.3389/fendo.2025.1587595)
Supplement: Supplementary file 1 [file DataSheet1.pdf]

The PCOS model was successfully established using C57BL mice.

Ovarian tissue HE staining:

One mouse was randomly selected from each of the PCOS group and control group for ovarian tissue HE staining. The results are shown in **Fig.5A** (observed at 100× magnification). Compared with the control group, the ovarian tissue structure of mice in the PCOS group was significantly abnormal, with irregularly shaped oocytes, a higher number of small follicles, and some atretic follicles. The granulosa cell layer surrounding the oocytes was significantly thinned, with some granulosa cells necrotic and missing, and red blood cells were present in the follicular cavity.

PCOS Mouse Estrous Cycle:

Microscopic examination of vaginal exfoliated cells was conducted starting from day 8 post-modeling to determine the estrous cycles of the two groups of mice. The characteristics of vaginal exfoliated cells during the normal estrous cycle are shown in **Fig.5B**. During the proestrus phase, vaginal exfoliated cells are primarily composed of partially nucleated keratinized epithelium; during the estrus phase, they are mainly composed of fully keratinized epithelium, almost entirely consisting of sheet-like, non-nucleated keratinized cells; during the metestrus phase, they are primarily composed of leukocytes and interstitial cells; during the anestrus phase, epithelial cells are scarce and shrunken, with a large number of leukocytes present in the smear.

Starting from the 8th day of modeling, vaginal smears were observed daily in both groups of mice, recorded, and plotted on a line graph. The results are shown in **Fig. 5C.** Compared with the control group, the PCOS group of mice exhibited irregular estrous cycles, characterized by prolonged estrous cycle duration and persistent estrus.

Weight changes in PCOS mice:

After one week of adaptive feeding, the mice were weighed once on the day the PCOS model was established, and then every three days thereafter until the end of modeling, for a total of seven weigh-ins. As shown in **Fig.5D**, the final body weight of mice in the PCOS group was higher than that of the control group, and the net weight gain of mice in the PCOS group was significantly higher than that of the control group (9.15 ± 0.87 g vs. 6.05 ± 0.68 g, **$p = 0.001$**). The results indicate that mice in the PCOS group had a higher body weight than those in the control group.

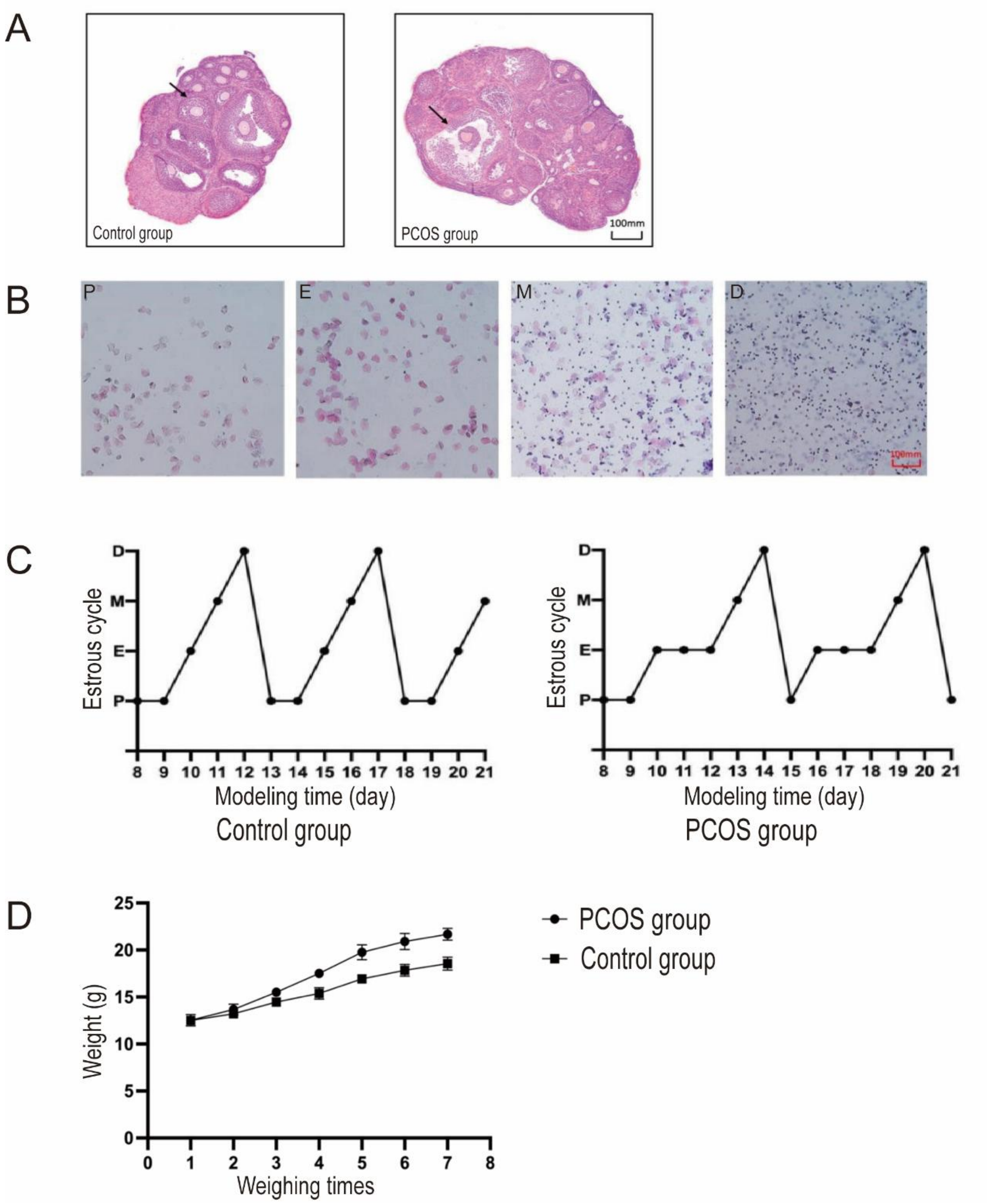

Figure. 5 The PCOS model was successfully established using C57BL mice. A Comparison of HE staining of ovarian tissue between the control group and the PCOS group. Black arrows indicate the morphology of individual follicles in the two groups. **B** Microscopic photographs of vaginal exfoliated cells during the normal estrous cycle of mice. P: proestrus, E: estrus, M: metestrus, D: diestrus. **C** Comparison of estrous cycles between the control group and PCOS group mice （Representative traces: C03, P08）. P: proestrus, E: estrus, M: metestrus, D: diestrus. **D** Changes in body weight trends among different groups of mice.

A daily stage overview table for 24 mice

P=proestrus

E=estrus

M=metestrus

D=diestrus

Day	D8	D9	D10	D11	D12	D13	D14	D15	D16	D17	D18	D19	D20	D21
Control														
C01	P	P	E	M	D	P	E	M	D	P	P	E	M	D
C02	E	M	D	P	P	E	M	D	P	E	M	D	P	P
C03	P	P	E	M	D	P	P	E	M	D	P	P	E	M
C04	M	D	P	E	M	D	D	P	E	M	D	P	E	M
C05	D	D	P	E	M	D	P	E	M	D	D	P	E	M
C06	P	E	M	D	D	P	E	M	D	D	P	E	M	D
C07	E	M	D	P	E	M	D	D	P	E	M	D	P	E
C08	M	D	D	P	E	M	D	P	P	E	M	D	P	E
C09	D	P	E	M	D	P	E	E	M	D	P	E	M	D
C10	P	E	M	D	P	E	M	D	D	P	E	M	D	P
C11	E	M	D	P	P	E	M	D	P	E	M	D	D	P
C12	M	D	P	P	E	M	D	P	E	M	D	P	P	E
PCOS														
P01	M	E	E	E	M	E	E	P	E	M	E	E	D	E
P02	E	M	M	E	E	E	P	E	M	E	E	M	E	D
P03	E	E	M	E	E	D	E	M	E	E	P	E	M	E
P04	M	E	E	P	M	E	E	E	D	E	M	E	E	M
P05	E	M	E	E	D	M	E	E	P	E	M	E	E	D
P06	D	E	M	E	E	M	M	E	E	P	E	M	E	E
P07	E	E	D	M	E	E	M	E	E	D	M	E	E	P
P08	P	P	E	E	E	M	D	P	E	E	E	M	D	P
P09	M	M	E	E	P	E	M	E	E	E	D	M	E	E
P10	E	D	M	E	E	E	M	P	E	M	E	E	M	D
P11	E	M	E	E	P	M	E	E	D	M	E	E	E	M
P12	D	M	E	E	E	M	P	E	E	M	M	E	E	D
